# The scale affects our view on the identification and distribution of microbial communities in ticks

**DOI:** 10.1186/s13071-020-3908-7

**Published:** 2020-01-21

**Authors:** Thomas Pollet, Hein Sprong, Emilie Lejal, Aleksandra I. Krawczyk, Sara Moutailler, Jean-Francois Cosson, Muriel Vayssier-Taussat, Agustín Estrada-Peña

**Affiliations:** 1 UMR BIPAR, Animal Health Laboratory, INRAE, ANSES, Ecole Nationale Vétérinaire d’Alfort, Université Paris-Est, Maisons-Alfort, France; 2 Centre for Infectious Disease Control, National Institute for Public Health and the Environment, Bilthoven, The Netherlands; 3 INRAE, Animal Health Department, Nouzilly, France; 4 Faculty of Veterinary Medicine, University of Zaragoza, Zaragoza, Spain; 5 Laboratory of Entomology, Wageningen University and Research Centre, Wageningen, The Netherlands

**Keywords:** Scales, Tick microbiome, Spatial, Temporal, Tick microbe interactions

## Abstract

Ticks transmit the highest variety of pathogens impacting human and animal health worldwide. It is now well established that ticks also harbour a microbial complex of coexisting symbionts, commensals and pathogens. With the development of high throughput sequencing technologies, studies dealing with such diverse bacterial composition in tick considerably increased in the past years and revealed an unexpected microbial diversity. These data on diversity and composition of the tick microbes are increasingly available, giving crucial details on microbial communities in ticks and improving our knowledge on the tick microbial community. However, consensus is currently lacking as to which scales (tick organs, individual specimens or species, communities of ticks, populations adapted to particular environmental conditions, spatial and temporal scales) best facilitate characterizing microbial community composition of ticks and understanding the diverse relationships among tick-borne bacteria. Temporal or spatial scales have a clear influence on how we conduct ecological studies, interpret results, and understand interactions between organisms that build the microbiome. We consider that patterns apparent at one scale can collapse into noise when viewed from other scales, indicating that processes shaping tick microbiome have a *continuum* of variability that has not yet been captured. Based on available reports, this review demonstrates how much the concept of scale is crucial to be considered in tick microbial community studies to improve our knowledge on tick microbe ecology and pathogen/microbiota interactions.
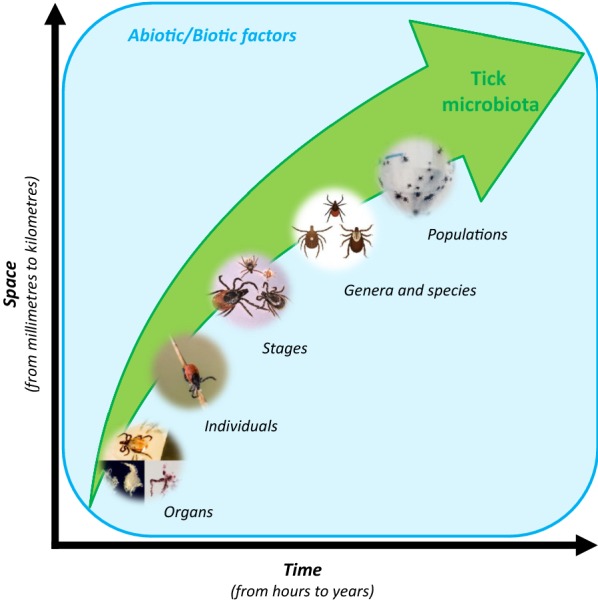

## Background

Ticks transmit pathogens of medical and veterinary importance. Their infections cause serious health issues in humans and considerable economic loss in domestic animals. Important steps in assessing disease risk and formulating possible intervention strategies involve understanding which factors drive population densities of ticks and the transmission dynamics of pathogens. Many environmental, landscape and anthropogenic factors are involved in determining the spread and abundance of ticks and transmitted pathogens. These factors are strongly interlinked and not yet well quantified. An additional layer of complexity is the tick microbiota, possibly affecting the fitness of ticks and consequently influencing their populations. Microbiota of ticks are ecological communities of commensal, symbiotic and parasitic microorganisms found in and on ticks. Tick-borne pathogens are artificially separated from the rest of the microbiota, based on a subjective human classification. Tick-borne pathogens (TBPs) are defined as microorganisms that are transmitted by ticks to a vertebrate and could cause disease. Nevertheless, pathogens can be considered as members of the tick microbiota, as they also have the ability to survive within ticks, change the fitness of tick populations and face the challenge of being transmitted from one tick generation to the next. Although pathogens are part of the tick microbiota, we will keep in this review distinguishing them from other microorganisms inhabiting ticks.

Thanks to various molecular approaches, epidemiological surveys have been performed to identify microorganisms, particularly bacteria, acquired and transmitted by ticks [1–30]. These reports increased our current understanding of TBPs epidemiology shifting from a “single” to a “multiple” pathogen view. For example, *Ixodes ricinus* is known to transmit more than 25 different pathogens affecting the health of humans or domestic animals: several studies have shown that one third of *I. ricinus* nymphs are infected with at least one pathogen and about 6% with more than one pathogen [31]. In addition, it is now well established that TBPs coexist with many other microorganisms (microbiota) in ticks constituting a tick microbial complex recently named pathobiome [32]. The microbial communities of several tick species of the genera *Ixodes, Dermacentor*, *Hyalomma*, *Haemaphysalis*, *Rhipicephalus* and *Amblyomma* have been studied [33–39] improving our knowledge on the diversity and composition of the tick microbiome. Microbiome often consists of endosymbionts, which can have multiple detrimental, neutral, or beneficial effects to their tick hosts [40, 41], and therefore might play various roles in fitness, nutritional adaptation, development, reproduction, defence against environmental stress, and immunity [42]. Otherwise, they may also contribute to transmission or multiplication of tick-borne pathogens [9, 32, 43], with many potential implications for both human and animal health. In this context, the identification and characterization of tick microbiota has become crucial to better understand tick-microbe interactions. With the development of high throughput sequencing technologies, the number of studies dealing with the tick pathobiome considerably increased in the past ten years revealing new pathogens and an unexpected microbial diversity in ticks [5, 39, 44–46]. It is of great interest to identify members of the microbial community which strongly affect the risk of TBPs transmission, either by affecting the fitness of ticks, their survival, or the transmission capacity of pathogens.

This microbiota is likely to vary according to the scale at which samples have been collected (spatio-temporal scale, the scale within the tick at organ level) or the context in which the study has been performed (environmental parameters). However, many studies dealing with tick microbes identified microbial communities in ticks without considering these scales that may therefore affect the interpretation of results. Consensus is lacking as to which spatial and temporal scales best facilitate understanding the role of tick microbial diversity and composition, and its potential influence on TBP transmission risk. Scales have a profound influence on how we conduct ecological studies, interpret results and understand the links between processes operating at different rates. All these factors deeply influence our ability to anticipate changes driven by the climate trends, ecological factors, environmental pollution, or antibiotic resistance on the tick microbiome. Patterns apparent at one scale can collapse to noise when viewed from other scales, indicating that perceptions of the importance of different processes vary in a scale-dependent manner. Moreover, the environment is not only an arena in which organisms can prevail, as they interact and alter the environment. The assemblages of human or animal TBPs, specific tick endosymbionts and other microbes (commensal or environmental) are likely to vary along with the geographical location or season, and can also depend on tick-related factors such as life stages and the anatomical location.

**Fig. 1:**
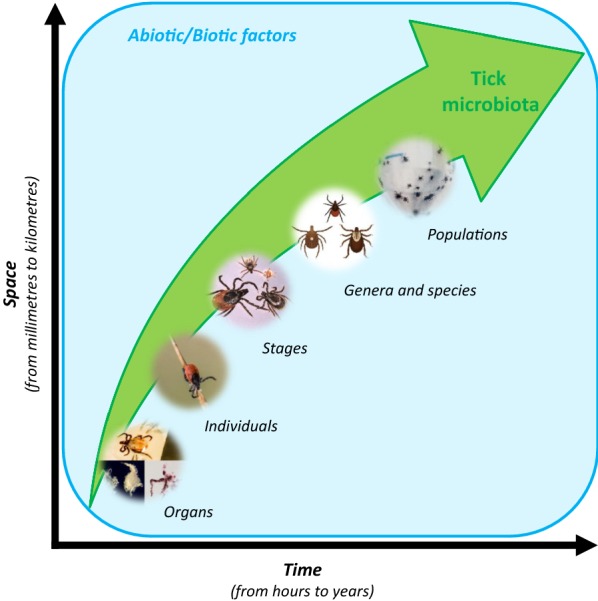
Improving our understanding of the tick microbial community ecology. Spatial and temporal studies ranging in scale from that of tick organs to population have allowed us detect patterns of distribution to finally develop predictive models on pathogen/microbiota interactions. Pictures: organs (Ladislav Simo), genera and species (CDC), populations (Philippe Garo, Agence Phanie)

This review aims to highlight the importance of working across multiple scales when dealing with tick microbial ecology. This integrated approach would allow to (i) better understand the ecology of tick microbial communities and their interactions; (ii) develop predictive models on pathogen dynamics and pathogen/microbiota interactions; and finally (iii) better understand tick-borne diseases. Spatio-temporal scales are just as important to consider as those defined at the level of the tick body (Fig. 1). Based on results from previous studies, we aim to demonstrate how the selection of scale influences our understanding of tick microbiome TBPs dynamics and microbiome-pathobiome interactions. This will be firstly addressed at the tick scales (organs *vs* whole tick body and the different tick stages) and then at the both temporal and spatial scales.

We are aware of the lack of a consensus about several definitions regularly used in tick microbial community ecology. The key definitions regarding tick microbes are provided in Table 1.

**Table 1:** Key definitions

Term	Definition
Tick pathobiome	Tick-borne pathogens in their microbial environment: tick-borne pathogens plus the rest of tick microbes potentially interacting with them
Tick microbiota	The assemblage of all microorganisms present in and on ticks
Tick microbiome	The collection of genes and genomes of members of the tick microbiota combined with the environment (Marchesi and Ravel [113])
Tick-borne pathogens	Microorganisms transmitted by ticks to humans or animals which have the ability to cause disease
Tick symbionts	Microorganisms engaged in close and long-term interactions with their tick hosts. They are required for tick survival and reproduction or have multiple effects on tick life history traits (Bonnet et al. [42]) Endosymbionts live inside tick; most of them have obligate intracellular life cycles and depend almost exclusively on maternal transmission

## The need to consider the different tick scales (organs, stages) to better understand the tick microbe ecology

### The organ scale

The new high throughput detection and sequencing approaches have revealed and identified a high diversity of TBPs. The vast majority of the reports focusing on pathogen detection have investigated whole ticks. However, it would be informative to look at the organ level due to the specific transmission dynamics of tick-borne pathogens. During blood-feeding, pathogens need to pass the barrier of the midgut (MG) to colonize the tick body, and the barrier of the salivary glands (SG) to be transmitted with saliva. Thus, despite that these are the primary organs for pathogen acquisition and transmission, few data are currently available on the organ-specific pathogen distribution. Comparing the numerous studies identifying pathogen presence and prevalence at the whole tick scale with the few of those performed at the tick organ scale [22, 47, 48], it is extrapolated that most of pathogens detected at the whole tick scale are logically found at a finer organ scale. Several contrasting findings can however be observed according to the tick scale we are looking at. With sensitive detection tools, tick co-infections are usually observed in tick-borne pathogen analyses [8, 29, 39].

Several studies on *I. ricinus* detected at the whole-body scale that the most common pathogen associations (positive or negative) were *Borrelia garinii* + *Borrelia afzelii*; *B. garinii* + *Borrelia lusitaniae*; and *B. garinii *+ *Borrelia spielmanii* [8, 12, 21, 49]. At the organ scale, it was noticed that the most common bacterial co-infections in male and female MG and SG were *Rickettsia helvetica + Anaplasma phagocytophilum* and *R. helvetica + B. lusitaniae*, respectively [22]. These contrasting results observed in pathogen associations thus depend on the tick scale (whole body *vs* organs). We detected different members of the complex *B. burgdorferi* (*sensu lato*) in both SG and MG of questing *I. ricinus* adults, which contrasts with the well-established postulate indicating that *B. burgdorferi* (*s.l*.) genospecies are not found in the salivary glands during the initial tick attachment, as they only move rapidly from the gut to the salivary glands at the beginning of the next blood meal. These findings suggest that some *B. burgdorferi* (*s.l*.) genospecies do not need a blood meal to start their multiplication and migration from the gut to salivary glands. This hypothesis has already been experimentally reported [50] showing that different *B. burgdorferi* (*s.l*.) strains were detected in female salivary glands before blood meal. These studies demonstrated how much it is crucial to consider both entire tick and organ scales to increase our current understanding of TBPs dynamics and ecology.

Most of studies describing the tick microbiota are based on the DNA extracted from whole ticks. Even if endosymbionts are mainly known to be vertically transmitted to the progeny *via* a transovarial transmission, multiple evidence suggests a probable horizontal transmission and possibilities to find them in other tick organs [43, 51–53] outlining a possible influence on pathogen acquisition and transmission. Despite this evidence, very few studies have identified the tick microbiomes at the tick organ scale [34, 54–59]. Interestingly, contrasting results in tick microbial communities are described according to the anatomical region within the tick. As an example, Gall et al. [58] identified microbes in the MG and SG of two *Dermacentor andersoni* populations reporting that the bacterial composition varied by organs. In the first studied population, the microbial community identified in MG had two endosymbiont groups, namely a mixture of *Francisella* spp. (20%) and *Francisella*-like endosymbiont (61%), whereas the SG microbiota was composed mainly of *Arsenophonus* spp. In the second tick population, the MG microbiota was primarily composed of *Francisella*-like endosymbiont (60%), *Francisella* spp. (20%), and a small proportion of a *R. bellii* (16%), whereas the SG microbiota was composed primarily of *R. bellii* (82%) and *Arsenophonus* spp. (11%). The detection of a large abundance of *Francisella*-like endosymbiont only in MG could be not surprising since this tick endosymbiont synthesises B vitamins that are deficient in the blood meal of ticks [41]. This observation is in agreement with another study [59] reporting that the majority of field-collected adult *I. scapularis* harbour limited internal microbial communities that are dominated by endosymbionts. Other results [60] suggest that *Coxiella* spp. and *Rickettsia* spp. are the main symbionts in three species of ticks, namely *Haemaphysalis longicornis*, *Rhipicephalus haemaphysaloides* and *Dermacentor silvarum*, symbionts primarily restricted to MG, Malpighian tubules and reproductive tissues; however, such tissue distribution varies in depending on species and sex. Clayton et al. [57] reported that the composition of the main endosymbionts changes over three generations in MG and SG of adult *D. andersoni* ticks. The endosymbionts included *Rickettsia*, *Francisella*, *Arsenophonus* and *Acinetobacter*, and their presence changed in contrasting proportions according to the considered organ.

In the case of *Francisella*-like endosymbionts, their presence in various tick organs is probably linked to a specific functional role that is still unclear. This hypothesis has been recently proposed [53] to explain the presence of *Midichloria mitochondrii* in different tissues inside the tick *I. ricinus*. These authors suggested that this primary *I. ricinus* endosymbiont could play multiple tissue-specific roles both enhancing tick fitness and/or ensuring its presence in the tick population. Epidemiological surveys and identification of microbial communities in ticks performed at the whole tick body are clearly relevant to characterize the presence and the dynamics of tick microbial communities. Identifying these microbes at the finer organ scale is probably more relevant with an aim of capturing the mechanisms of pathogen transmission and the potential influence of the tick microbiome in these mechanisms. Nevertheless, it should be noted that this approach can be particularly difficult to perform due to the limitations of manipulating tick organs. Dissection of adult ticks is indeed possible, but translating the method to nymphs is a challenge. In addition, it is time consuming and it is necessary to be careful during the organ extraction to avoid potential cross-contaminations.

### Tick stages: larvae *vs* nymphs *vs* adults

Ticks of the family Ixodidae undergo either one-, two- or three-host life-cycles. Most ticks of public health importance undergo a three-host life-cycle (larvae, nymphs, adults), in which after a blood meal, a tick leaves its host for moulting or egg-laying. Ticks potentially acquire pathogens from hosts during their different blood meals. A high density of hosts might hypothetically drive larger possibilities to acquire TBPs. The prevalence of TBPs in *I. ricinus* nymphs and adults in pastures and woodlands in France has been already studied [2]. Results showed that the highest infection prevalence of *B. burgdorferi* (*s.l*.), *A. phagocytophilum* and *Rickettsia* spp. was found in adult females. The prevalence of *B. burgdorferi* (*s.l*.) in nymphs was lower than 6%. Contrasting results were observed [12] in three geographically distinct areas of eastern Romania where the estimated prevalence of eight *Borrelia* species was very similar between adults and nymphs. Similar results have been already confirmed for *I. ricinus* [61] over a three-year survey in a peri-urban forest in the south of Paris, France. Strnad et al. [49] reported that the overall prevalence of *B. burgdorferi* (*s.l*.) in adult ticks was higher than in nymphs and among adults, prevalence was higher in females than in males. This last result matches our recent observations where we evaluated the presence of pathogens in SG and MG in both *I. ricinus* males and females, and detected *B. lusitaniae*, *B. spielmanii* and *B. garinii* only in females [22]. In Switzerland, *B. valaisiana* and *B. spielmanii* had significantly lower prevalence in *I. ricinus* males than in females [1]. In another study, it has been demonstrated that bunyaviruses are widely distributed and abundant in both *Ixodes scapularis* males and females [55]. While mean prevalence for one or several pathogens are commonly estimated “in toto”, these contrasting results raise the importance of analyzing the tick stages separately, calculating the prevalence for each stage. Examination of all potential ways of pathogen maintenance and transmission throughout the vector-pathogen life-cycle will help to understand the epidemiology of tick-borne pathogens [62].

Tick activity, metabolism and physiological functions are likely to vary between tick stages. Because tick endosymbionts are likely to have a crucial role on nutrition, fitness, development, reproduction, defence against environmental stress, and immunity in the tick life-cycle [42], it could be easily hypothesized that passing through stages could potentially influence the tick microbiome. As previously mentioned [44], it is highly conceivable that the maternal microbiota might serve as the first inoculum in eggs and larvae. It is necessary to consider the probable role of the environment in the acquisition of the first microbiota because these ticks hatched in a sterile environment [56]. It seems that the microbiome might then change according to the different stages and the tick sex. For “generalist” ticks, feeding on a large number of vertebrates, the most obvious hypothesis to explain differences in the microbiomes between tick stages would be that the host blood meal would have a strong impact on microbiome species richness and composition, as already observed on *I. pacificus* [45]. However, other studies have not found correlations with host blood [63, 64] even if authors suggested that the very high proportion of tick-specific endosymbionts might mask the effects of the blood of vertebrate hosts [63]. More investigations are thus necessary to clarify this point. Studies about different tick species pointed out that the taxonomic diversity indices of the microbiome estimated for males were significantly higher than those estimated for females [34, 65, 66]. As suggested in these studies, microbiome in females was probably less diverse because they had higher relative burdens of *Rickettsia* and a highly dominant endosymbiont. In the same way the microbiome of *I. pacificus* across life stages shows a decrease in both species richness and evenness as the tick matures from larva to adult [67]. Please note that for most of these studies, no controls have been performed to remove potential contaminants. The hypothesis that these studies may have been biased by detecting contaminant bacteria, coming from both extraction and amplification steps should thus not be ruled out [68]. In any case, studies recurrently support the differences of the tick microbiota according to tick life stages [63, 65, 66, 69, 70].

For ticks collected in the same area, variations in microbial community composition between stages is likely to be shaped by stage-specific endosymbionts. Females of *R. sanguineus* (*s.l*.) collected in different regions in France had a microbial community dominated by *Rickettsia* or *Coxiella* while these symbionts were rarely detected in nymphs or males [69]. Similar results were observed for *I. scapularis* [66]. *Midichloria mitochondrii* is detected in many tick species [71] and shows nearly 100% prevalence in females of *I. ricinus* while it is much less prevalent, even absent, in males [72, 73]. This kind of information is crucial as recent studies suggest that the presence of *M. mitochondrii* could influence the growth of the spotted fever group rickettsial agent, *Rickettsia parkeri*, in the tick *Amblyomma maculatum* [43]. The presence of certain endosymbionts could otherwise influence the development of ticks and this role should be investigated in the near future. *Arsenophonus* spp., which are symbionts detected in several tick species [74–76] are indeed known to be responsible for sex-ratio distortion in arthropods, and some studies suggest that they can affect host-seeking success by decreasing tick motility in *A. americanum* and *D. variabilis* [77]. However, it is necessary to remain cautious about the role of *Arsenophonus* spp. as tick symbionts since it has been suggested [78] that *Arsenophonus nasoniae* in ticks may not originate directly from a tick but from its parasitizing wasps, *Ixodiphagus hookeri*. Similarly, some *Spiroplasma* spp. already detected in *Ixodes* spp. such as *Spiroplasma ixodetis* [79] are known to cause sex-ratio distortion in some insect species *via* male killing [80]. The role of all these endosymbionts still remains unclear in ticks and have to be investigated. Considering the very variable prevalence of *M. mitochondrii* in females or males of ticks, could its presence/absence in nymphs influence the future male and female differentiation? While we know that endosymbionts could supply benefits to ticks with a potential role in tick development, reproduction, moult or pathogen acquisition and transmission, the question is “which taxa are doing what”. Investigating different life stages and sexes could help to answer and infer these roles.

This first part demonstrated how much it is crucial to consider the different tick scale in tick microbial community studies to increase our current understanding of tick microbe dynamics and ecology. Both diversity and composition of tick microbial communities are highly variable and environmental constraints might be key drivers of their structure. A better control of ticks and TBPs especially requires answering what external factors shape the tick microbial communities. For that, studies should not be restricted to report a list of bacterial taxa but investigate tick microbial communities in a more ecological context considering both the spatial and temporal dynamics of these communities.

## The need to consider both spatial and temporal scales

### Temporal scales

#### Do transmission cycles of tick-borne pathogens have a temporal scale?

**Fig. 2:**
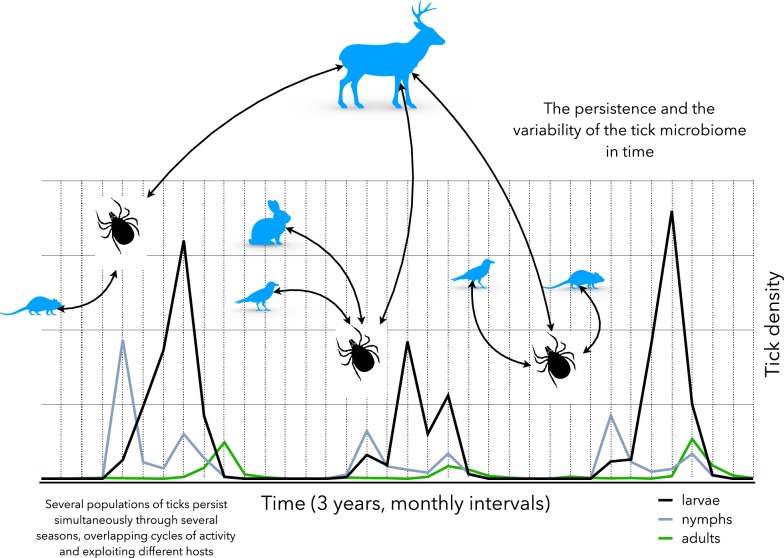
The time scale may affect the composition of tick microbiome. The illustration schematically represents the seasonal and inter-year variations of a hypothetical population of ticks. The different stages of a tick species have different inter-year densities and a variable seasonality in the same territory that is mainly caused by climate factors. The three stages of the tick may coexist at the same time and at the same habitat patch, and their dynamics (as lines in the figure) may differ from year to year. According to the moment of the year and the host availability, ticks can exploit different species of hosts, resulting in an “exchange” of bacteria obtained from blood meal that may be incorporated into the gut microbiome of the ticks. While immature stages may feed on small vertebrates, large ungulates can also support large numbers of immatures and adults. This adds variability to the microbiome because the seasonality is different each year. Climate shapes these patterns and host availability is different at different moments of the year. The X-axis represents three years and the Y-axis indicates tick density (hypothetical values). Silhouettes of vertebrates and ticks are merely illustrative and do not represent a specific vertebrate or tick stage

The span of the life-cycle of a tick is highly variable, with the exception of the one-host ticks (i.e. some species of the genus *Rhipicephalus*) or the genus *Otobius* (Fig. 2). Most ticks quest or ambush for substantial amount of time before finding an adequate host. Also, temperature and diapause play a pivotal role in the duration of the moulting and questing periods [81]. All these factors deeply affect the total duration of one generation of a species of tick. For example, the life-cycle of *I. ricinus* may last for 2–3 years in its distribution range [82] or up to 5 years in colder regions. Similar values have been reported for the closely related species *I. scapularis* and *I. persulcatus* [83]. This variability in the tick life-cycle results in a variable length of contacts among ticks and the reservoirs of pathogens or even a possible lack of overlap according to the seasonality of both the tick and vertebrate populations. Since the ticks may overwinter, resuming the questing activity the following spring, the same generation could potentially feed upon different generations of hosts, resulting in a kind of looping cycle re-infecting hosts that are newly incorporated to the populations of reservoirs. In the same way, hosts surviving the winter may infect a population of ticks with “new” pathogens, resulting in variable prevalence rates and co-infections patterns. However, these cycles of transmission *in time* have not yet been studied in detail with the exception of local studies, focused on certain associations of ticks and vertebrates. We hypothesize that such turnover between hosts and tick populations may potentially lead to a large variability of the tick microbiome, resulting in variable associations ticks-microbe in short periods of time.

An interesting pattern of longevity and therefore of the long persistence of pathogens in the habitat is that of ticks of the family Argasidae. Most species have long-lasting generations, even if they feed only for some minutes, as a consequence of their particular habitat inside the shelter of a small vertebrate (i.e. Rodentia or Soricomorpha). Some species of the genus *Ornithodoros* (Argasidae) may have as many as 9 nymphal stages and each one can live for a variable period. Commonly this period ranges between 6–9 years [84, 85], a trait that strongly depends on the availability of hosts in the shelter in steppe or desert areas in which hosts density is very low [86]. Therefore, these ticks could be infected by different microbes carried by consecutive generations of reservoirs. Our hypothesis is that this longevity associated with different generations of hosts would result in complex patterns of bacterial co-existence in the otherwise very spatially restricted population of ticks. These spatial restrictions derive from the nidicolous life-style of the *Ornithodoros* ticks driven by the patchy nature of the natural territory of their rodent or bird hosts. Since these ticks feed for a short time, while hosts are resting in the burrow, the probabilities of exchange of populations are low. This would most probably give rise to a changing pattern of microbial prevalence rates, not only spatially, but over time, an extreme that has never been addressed.

Regarding other better studied ticks, like species of the genus *Ixodes*, it is necessary to capture the life span of the many reservoirs of the circulated pathogens. While the life span of rodents or soricomorphs is typically short, birds can live for several years, the life span commonly being in a direct relationship with size. Carnivores and large ungulates tend to live at least one decade. This provides a particularly puzzling background over which the tick generations interact with infected vertebrates having different life spans. If a vertebrate can live longer, it has larger probabilities to be bitten by ticks. The background of this patchy transmission pattern in time reveals profound differences between some pathogens, like *Anaplasma phagocytophilum*, that can be reservoired by large ungulates, or *Borrelia* spp., that are commonly circulated by small mammals and birds. For *Borrelia* spp., the ability of using many species of animals with different life spans seems to be an optimal strategy to infect multiple generations of ticks, irrespective of their stage. Since several vertebrates are involved in the circulation of the pathogen [87], the three-host ticks, like *I. ricinus*, *I. persulcatus* or *I. scapularis*, can be infected at any stage of their life-cycle. The attachment of *A. phagocytophilum* to such large variety of hosts ensures that the pathogen can circulate through short duration cycles (in rodents-ticks) while the large ungulates support a long-lasting cycle. These short-term transmission cycles could deeply operate on the speciation and segregation processes of strains of pathogens like *A. phagocytophilum*, giving phenotypic flexibility to the populations of the pathogen while the long-term transmission cycle (supported by large ungulates in this pathogen) would allow the persistence of a long-standing genetic background, ensuring the durability of its “basic genetic traits”. On the other hand, pathogens like *Borrelia* spp. have also different time windows of transmission along the seasonal activity of both small mammals and birds. Summarizing this view, the annual pattern of activity of both ticks and reservoirs of pathogens, may lead to unsuspected patterns of variability in the tick microbiome (or pathobiome) because of loops of short- and long-term transmission cycles, depending upon the expected survival age of the reservoir.

#### Is the temporal scale crucial to study the tick microbiome?

As previously mentioned, over their life-cycle, ticks are likely to experience the influence of the temporal variation of multiple factors such as temperature, hydric stress and diapause. All these factors are known to probably influence tick activity and metabolism and might potentially affect their microbiome [70]. While studies on tick microbial community diversity, composition and role have considerably increased in the past years, many questions arise about the temporal dynamics of the tick microbiome. Do the tick microbial diversity and composition depend on the temporal scale? Is the temporal scale (season, multi-year surveys) crucial to study the tick microbiome to allow acquisition of more information about tick microbiome? These questions are particularly relevant because some tick symbionts are involved in the tick activity and metabolism [41] and in the tick-borne pathogen acquisition and transmission [43, 45]. Are these functions constant all over the tick lifetime making endosymbiotic presence constant as well, or are bacterial communities of a dynamic nature? Moreover, as observed for mosquitoes [88], blood ingested by ticks is rich in proteins and lipids and probably digested by gut microbial communities. While blood meals represent a short part of the tick life-cycle, what is the temporal dynamics of these microbes? Do their relative composition and abundance change during the questing period? Are they replaced by other microbial communities involved in other metabolic functions? Unfortunately, it is premature to answer categorically all these questions due to the limited information available about the temporal dynamics of the tick microbiome. To the best of our knowledge, only Lalzar et al. [89] conducted a brief temporal survey on tick microbes collecting weekly two *Rhipicephalus* tick species from March to July in Israel. They showed that the bacterial community structure of *Rh. turanicus* was characterized by high dominance of *Coxiella* and *Rickettsia* and exhibited extremely low taxonomic diversity. *Coxiella* spp. densities were overall stable throughout the questing season while *Rickettsia s*pp. significantly declined toward the end of the questing season.

The variability in the tick life-cycle and particularly the moment of the questing activity, the blood meal, the moult or the egg-laying, is affected by different factors: the density and type of hosts, and environmental factors such as temperature and hygrometry. All these factors vary through the seasons and ticks have to regularly face up to these variations. Moreover, while it is now well admitted that the pathogen dynamics in ticks is highly variable throughout the year with a contrasting seasonal prevalence [18, 21, 31, 90–92]), no long term studies are available to identify potential seasonal or annual patterns in tick microbiome and evaluate the impact of all these factors on microbial communities. It is crucial to fill the gap of knowledge about the temporal dynamics of the tick microbiome. These data would illustrate the temporal patterns in tick microbial community diversity and composition, and the potential redundancy of these patterns from one year to another.

### Spatial scales

#### What is the spatial scale of a tick and the associated pathogens?

**Fig. 3:**
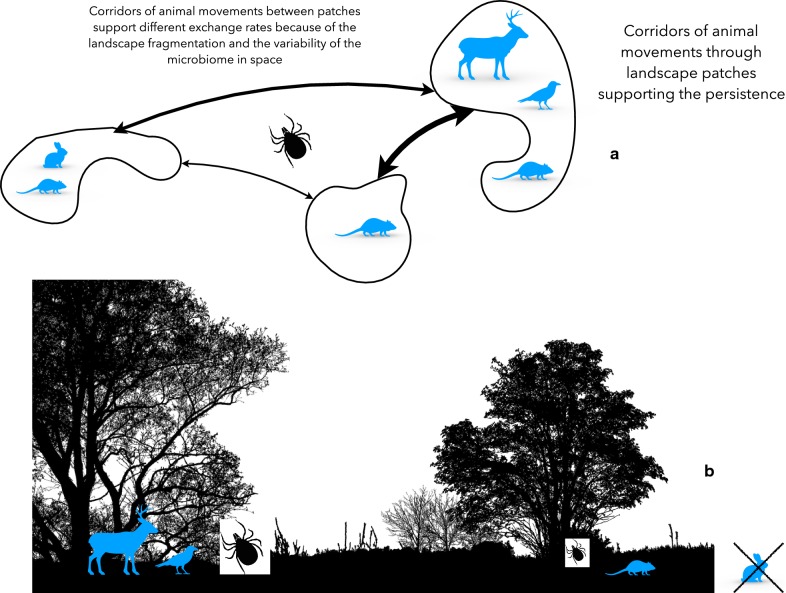
The spatial scale as driver of variability in the tick microbiome. **a** The connectivity patterns of the landscape drive the presence/absence of some key hosts and exchange of animals among patches, shaping a variable host composition in each patch. Some patches may be highly connected (wide arrows) while others are poorly connected, blocking the movements of vertebrates. This adds a spatial component to the composition and the variability of the tick microbiome. **b** The vegetal composition of the habitat may differ and modulate the microclimate, shaping tick density. The figure intends to show a gradient of biomes, in which hosts may be abundant or scarce, or even absent. Ticks also have different survival and questing rates at the small scale of the habitat patch. Such intra-patch spatial diversity shapes an extra variability of the tick microbiome

It has been repeatedly reported [93, 94] that ticks interact with their hosts at critical scales of the landscape. It is believed that ticks and hosts overlap in portions of the environmental niche that drive the rates of contact among the ticks and the adequate reservoirs for TBPs. Therefore, the effects at the *local* scale of a tick population are observed at the patch where these organisms co-exist. Ticks depend on the movements of vertebrates (i.e. birds or ungulates) to be able to prevail as a meta-population. These hosts move and spread ticks in a gradient along the patches of the habitat [95, 96]. It is important to realize that the structure of the habitat and the corridors of connectivity among the patches of the landscape are recognized as important features driving the survival of ticks as permanent populations at a site (Fig. 3).

The effects of the structure of the landscape on the presence/absence or the density of ticks are of particular importance in outlining the prevalence of TBPs in ticks [97]. Not only the different combination of vegetation categories outline the density of prominent reservoirs, but also the connectivity among the patches of landscape is an important trait [98]. Connectivity explains the effect of the spatial composition of suitable patches of vegetation that delineates a matrix of connections among these patches. These corridors explain the routes that mammals and birds use to move across the matrix of suitable and unsuitable habitat [99]. Therefore, a specific type of vegetation may be suitable for a high abundance of a given reservoir, therefore feeding ticks that could result infected with TBPs carried out by such reservoir [100]. However, the movements of the reservoirs across the matrix of the landscape patches could explain the variable rates, at the micro-spatial scale, of the prevalence of pathogens. Some habitats are prone to sustain different combinations of reservoir host (in both presence and density) and therefore this is reflected at the bacterial prevalence in the tick. In sites in which a prominent reservoir may be less abundant, a combination of other vertebrates could keep prevalence at comparable levels. This is the main new paradigm that has evolved from several field studies on TBPs [101–104]. The specific density of different vertebrates with a variable reservoir ability shape the prevalence of TBPs at small scales [105]. Even without the existence of geographical barriers (like a river or a hill that could prevent the spread of terrestrial vertebrates), the prevalence is very local in nature [106] and tends to distort the results about microbiome/pathobiome when explored at larger scales.

Studies from field surveys allowed the emergence of a paradigm for understanding the spatial scale of the tick pathobiome: the macro-climate regulates a continental pattern of contact rates among ticks, hosts and reservoirs that may be adequately quantified [107, 108], while micro-climate and the structure of the landscape shape such contact rates along peculiar patterns. These statements introduce another important concept: do different TBPs have contrasting critical spatial scales of persistence? How are pathogens affected by the local structure of the landscape, the resulting micro-climate and the movements of hosts? While studies exist about the importance of special configurations of the landscape on the movements of vertebrates, terrestrial animals and birds, no studies demonstrating how these movements alter the local prevalence of pathogens in ticks seem to exist. For example, it could be possible that the spatial scale of *B. garinii* (reservoired by birds) is larger than that of *B. afzelii* (reservoired by small mammals) since the movements of the former are less affected by local landscape structures. However, it is difficult to disentangle the movements of i.e. birds in different seasons of the year, carrying different stages of ticks, and spreading at different rates through the matrix of suitable habitat. We envisage a fruitful field of study in the understanding of these movements and their effects on the local composition of the tick pathobiome.

#### Is the spatial scale necessary to efficiently study the tick microbiome?

As previously mentioned, the concept of spatial scale is likely to be highly linked to environmental niche (landscape topography, climatic factors, vegetation) in which ticks and hosts evolve. Is the tick microbial diversity and composition influenced by spatial scale? Which spatial scale is the best investigation of the tick microbiome? Field studies have shown the strong influence of biogeography at large scales (states or regions) on the tick microbiome structure and composition on different tick species, *I. scapularis* [66], *I. ricinus* [109–111] and *A. americanum* [112]. For the same tick species, noting variations in tick microbiome composition and diversity at large spatial scales (between ticks collected from different states or regions) could not be so surprising due to the high variations in climatic factors and environmental niche. Contrasting results were recently observed as Clow et al. [37] have shown the lack of significant differences in the relative abundances of microbial communities of ticks collected in distant locations (east *vs* south) in Canada. However, these observations were made on relatively small sample sizes.

While contrasting biogeographical patterns were thus generally observed at large scale, more questions are emerging about tick microbial communities at finer scales. Do tick microbial communities have a “small” (local) spatial scale? Are tick microbial communities influenced by different local structures of the landscape, the local vegetation and the movements of hosts? We recently performed a study with the aim to build a network-based framework for analyzing co-occurrence patterns of microorganisms in *I. ricinus* ticks and one of its main hosts, the vole *Myodes glareolus* collected in two close but different ecosystems, Forests *vs* Ecotones (i.e. the edge networks within open grasslands) [46]. Results revealed that the microbiome of *I. ricinus* varied between ticks collected in forests and those collected in ecotones and that the local biotope could play an important role in shaping the bacterial communities of ticks. From these studies, it appears obvious to conclude that the spatial scales (from the largest to the most local scales) at which tick microbes are studied generally affect the tick microbiome diversity and composition. Biogeographical aspects, especially environmental niche should be even more considered in future tick microbial community studies to better identify factors that shape tick microbial communities and tick-borne pathogens. Combining studies at both large and local spatial scales would allow identifying the maximum of factors influencing tick microbial communities and include them in future predictive models to better understand tick microbe ecology.

## Conclusions

The story scientists are reporting makes sense only if the story is considered in a context. Moreover, this story can be understood by the reader only in the context in which the story is related. Based on examples from the previous research, we tried to show how much the concept of scale and the ecological context are crucial in studying tick microbial communities: first, to improve our knowledge on tick microbe ecology and secondly to facilitate successful strategies to control tick-borne diseases. All findings presented in this review clearly show contrasting and informative results according the tick stages and anatomical structures or the spatio-temporal context. This highlights the importance of considering all these different scales to study tick microbial communities and represents another step towards improved understanding of TBP transmission and tick microbe ecology. Listing and providing prevalence data of tick microbes has been an important step towards identifying pathogens transmitted by ticks and understanding the microbial complexity associated with ticks. All these data are essential but without considering scales and the environmental context in which ticks evolve, their use to better understand ticks and tick-borne diseases remains limited. Some important key points: (i) Patterns we observe depend on the scale at which they are studied; (ii) How organisms interact with the environment depends on the scale at which this interaction is studied; (iii) Because most processes are scale-dependent, we have to explicitly consider the scale in study design; (iv) Identifying a maximum of environmental factors potentially influencing tick microbes requires combining multiscale studies.

## Data Availability

The datasets used and/or analysed during the present study are available from the corresponding author upon reasonable request.

## Abbreviations

TBPs: tick-borne pathogens
*s.l.*: *sensu lato*
MG: midgut
SG: salivary glands
